# Season of birth effect on psychotic-like experiences in Japanese adolescents

**DOI:** 10.1007/s00787-012-0326-1

**Published:** 2012-09-17

**Authors:** Mamoru Tochigi, Atsushi Nishida, Shinji Shimodera, Yuji Okazaki, Tsukasa Sasaki

**Affiliations:** 1 Department of Neuropsychiatry, Graduate School of Medicine, University of Tokyo, 7-3-1 Hongo, Bunkyo, Tokyo, 113-8655 Japan; 2 Department of Psychiatry and Behavioral Sciences, Tokyo Metropolitan Institute of Medical Science, Kamikitazawa 2-1-6, Setagaya-ku, Tokyo, 156-8506 Japan; 3 Department of Neuropsychiatry, Kochi Medical School, Kohasu Oko-cho, Nankoku, Kochi, 783-8505 Japan; 4 Tokyo Metropolitan Matsuzawa Hospital, 2-1-1 Kamikitazawa, Setagaya, Tokyo, 156-0057 Japan; 5 Department of Health Education, Graduate School of Education and Office for Mental Health Support, University of Tokyo, 7-3-1 Hongo, Bunkyo, Tokyo, 113-8655 Japan

**Keywords:** Schizophrenia, Winter birth, Summer birth, Hallucination, Delusion

## Abstract

A number of studies have investigated seasonality of birth in schizophrenia. Most of the studies have consistently observed an excess of winter births, often associated with decreased summer births. We postulated that psychotic-like experiences (PLEs), subclinical hallucinatory and delusional experiences, may also be affected by birth season. In the present study, we assessed the season of birth effect on the prevalence of PLEs using data from the cross-sectional survey of 19,436 Japanese adolescents. As a result, significant excess of winter births was observed in the prevalence of PLEs, accompanied by a decreased proportion of summer births. The odds ratios for the prevalence of PLEs were estimated to be 1.11, which was on the same order with those for the development of schizophrenia in the previous meta-analytic studies. To our knowledge, this is the first to show the seasonality of birth in the prevalence of PLEs and implicate the winter birth effect on subclinical stage of schizophrenia.

**Electronic supplementary material:**

The online version of this article (doi:10.1007/s00787-012-0326-1) contains supplementary material, which is available to authorized users.

## Introduction

A number of studies have investigated seasonality of birth in schizophrenia. Most of the studies have consistently observed an excess of winter births, often associated with a decreased summer births [1–3]. The rate of the increase of winter births was around 5–15 %, compared with the expected number of birth during the season, in most of the Northern Hemisphere [1]. The odds/relative risk ratios of winter births for the development of schizophrenia have been estimated in the order of 1.10 [1–3]. Various hypotheses to explain the reason for the seasonality have been discussed, including meteorological variables, infections, maternal hormones, sperm quality, nutrition and external toxins, although the discussion has not reached conclusion [4].

Psychotic-like experiences (PLEs) are subclinical hallucinatory and delusional experiences, occurring not only in psychotic patients but also in a portion of the general population [5, 6]. Recent studies have suggested that PLEs also occur in children and adolescents [7–9], and a continuum between PLEs in childhood and schizophrenia spectrum disorder in adulthood was demonstrated [10]. Although the predictive validity of PLEs for psychotic disorder remains to be further studied, especially in case of those assessed by self-rating questionnaires [11], it may be worth investigating this phenomenon to elucidate the etiopathological mechanisms of schizophrenia and establish strategies for its prevention.

PLEs share an extensive range of risk factors with schizophrenia [12], while an association with winter–spring birth has failed to be shown [13]. Considering the previous study [13] used a total of 2,232 subjects, we may expect to detect the significant effect of birth season using larger amount of subjects. In the present study, we assessed the seasonality of birth effect on the prevalence of PLEs using data from the cross-sectional survey of 19,436 Japanese junior high and high school students.

## Subjects and methods

### Subjects

We used data from the cross-sectional survey of psychopathologies conducted from 2008 to 2009 in Kochi and Mie prefectures, Japan. Both prefectures are located in the mid to west part of Japan and approximately 300 km apart from each other, including both urban and rural areas (populations are approximately 750,000 and 1,800,000 for Kochi and Mie, respectively). In this survey, data were collected from students from 45 public junior high schools (7th–9th grade) and 28 senior high schools (10th–12th grade). Most of the schools in the middle areas of the two prefectures agreed to cooperate, and all of the schools were public. The total number of the current students of those high schools was 19,436 at the survey, and all of them were ethnically Japanese. Details of the procedure were described elsewhere [14]. We complied with Japan’s Ethical Guidelines for Epidemiological Research, and the study was approved by the ethics committees of the Tokyo Institute of Psychiatry, the Mie University School of Medicine, and the Kochi Medical School.

### Measures

PLEs were assessed by self-rating questionnaires using four items adopted from the schizophrenia section of the Diagnostic Interview Schedule for Children (DISC-C) [15]. These items were previously used in a birth cohort study and good predictors of schizophrenia spectrum disorder in adulthood [10]. The items were (1) “Some people believe that their thoughts can be read. Have other people ever read your thoughts?” (thoughts read); (2) “Have you ever had messages sent especially to you through the television or radio?” (special messages); (3) “Have you ever thought that people are following you or spying on you?” (spied upon); and (4) “Have you ever heard voices that other people cannot hear?” (heard voices), with a choice of three responses, ‘no’, ‘yes, likely’, or ‘yes, definitely’. We defined ‘yes, definitely’ as the presence of a hallucinatory and delusional experience and ‘no’ or ‘yes, likely’ as no experience.

### Statistical analysis

First, we defined winter and summer months according to the ambient temperatures (Supplementary Table 1) [16]. Winter months was defined as November to March, the average lowest temperature in the past 20 years of which was lower than 10 °C. Summer months was defined as July to September that of which was higher than 20 °C. April to June and October were treated as other months. We then assessed the seasonality of birth effect on the prevalence of PLEs using the Cochran–Armitage test for trend, i.e., the ordinal variables were allocated to winter, other, and summer months, and the linearity between the variable and the prevalence of the experience of at least one type of PLEs, “heard voice”, or “spied upon” was tested. “Heard voice” and “spied upon” were analyzed separately on the basis of two reasons: one is that they may be considered as continua of delusion and auditory hallucination, typical positive symptoms of schizophrenia [6]. The other is that when using a self-report questionnaire, the sensitivity and specificity of these two items were among the highest to screen PLEs in adolescents in the previous study [17]. Last, we estimated the odds ratios of winter/summer births for the prevalence of PLEs using Chi-square test. The prevalence of the PLEs, “heard voice”, or “spied upon” was compared in winter versus summer and other months, or summer versus winter and other months in all and divided subjects by gender, age (junior/senior high school), and survey area.

## Results

Out of 19,436 students of the 45 junior and 28 senior high schools, 798 students (4.1 %) were absent on the days of the survey, and 388 students (2.0 %) did not agree to participate in the study. Thus, the total of 18,250 students (93.9 %) answered the questionnaire. Out of 18,250 subjects, 548 were excluded due to missing data for PLEs. Consequently, 17,702 subjects [8,747 males and 8,955 females, age = 15.2 ± 1.7 years (mean ± SD.)] were analyzed.

The prevalence of the four PLEs was as follows: “thoughts read” was observed in 205 subjects (1.2 %), “special messages” in 131 (0.7 %), “spied upon” in 1,141 (6.4 %), and “heard voices” in 1,715 (9.7 %) (the distributions by birth months are shown in Supplementary Fig. 1). The experience of at least one type of PLEs was reported by 2,540 (14.3 %); 575 students (3.2 %) experienced two or more symptoms of PLEs.

**Fig. 1:**
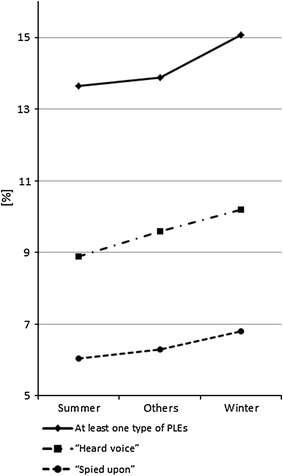
Prevalence of PLEs according to the birth seasons: summer (July–September), others (April–June and October), and winter (November–March)

Figure 1 summarizes the prevalence of PLEs according to the birth seasons. A significant association between the birth seasons and the experience of at least one type of PLEs or “heard voice” was observed (χ^2^ = 4.24 and 5.54, *df* = 1, *p* = 0.022 and 0.019, respectively).

As shown in Table 1, the odds ratio of winter birth excess was statistically significant in the experience of at least one type of PLEs and “heard voice” (OR = 1.11 and 1.11, 95 % CI = 1.02–1.21 and 1.00–1.23, *p* = 0.016 and 0.042, respectively). That of summer birth deficit was statistically significant in “heard voice” (OR = 0.85, 95 % CI = 0.79–0.99, *p* = 0.041). After dividing the subjects by gender, the odds ratios of winter birth excess and summer birth deficit were statistically significant in the experience of at least one type of PLEs in females (OR = 1.13 and 0.88, 95 % CI = 1.01–1.27 and 0.77–1.00, *p* = 0.030 and 0.048, respectively), while not in males (Supplementary Table 2). With respect to age, the junior high school students showed the statistically significant level of odds ratios for winter birth excess in the experience of at least one type of PLEs and “heard voice” (OR = 1.14 and 1.20, 95 % CI = 1.02–1.29 and 1.04–1.37, *p* = 0.026 and 0.010, respectively) (Supplementary Table 2). After dividing the subjects by survey area, the subjects in Mie prefecture showed the statistically significant level of odds ratios for winter birth excess in the experience of at least one type of PLEs, “heard voice”, and “spied upon” (OR = 1.31, 1.37 and 1.27, 95 % CI = 1.13–1.54, 1.14–1.64, and 1.02–1.58, *p* = 0.00054, 0.00073 and 0.031, respectively). The Mie subjects also showed the statistically significant level of odds ratio for summer birth deficit in the experience of “heard voice” (OR = 0.78, 95 % CI = 0.63–0.97, *p* = 0.025). In the Kochi subjects, the effect did not reach statistical significance (Supplementary Table 2).

**Table 1:** The odds ratios of winter/summer births for the prevalence of PLEs

	At least one type of PLEs	“Heard voice”	“Spied upon”
Winter	1.11 (1.02–1.21)**	1.11 (1.00–1.23)*	1.11 (0.98–1.25)
Summer	0.93 (0.84–1.02)	0.85 (0.79–0.99)*	0.91 (0.79–1.05)

Odds ratios were calculated by comparing winter versus summer and other months, or summer versus winter and other months (described with 95 % CI in the brackets)

* *p* < 0.05

** *p* < 0.02

## Discussion

The present results showed a significant excess of winter births in the prevalence of PLEs in the Japanese adolescence, accompanied by a decreased proportion of summer births especially in “heard voice”. The odds ratios for the prevalence of PLEs were estimated to be 1.11, which was on the same order with those for the development of schizophrenia in the previous meta-analytic studies [1–3]. This may be the first study to show the seasonality of birth in the prevalence of PLEs and suggest the winter birth effect on subclinical stage of schizophrenia.

The present subjects derived from Kochi and Mie prefectures, having mid to small size of population and located within the not far distance in the mid to west part of Japan. All of the recruited schools were public and distributed in the middle areas of the two prefectures, including urban and rural areas. Therefore, it is unlikely that the data of the subjects are significantly deviated from that of the general population of Japan in this generation. In the analysis after dividing the subjects by gender, age (junior/senior high school), and survey area, female, junior high school, and Mie subjects showed the statistically significant odds ratio of winter birth excess and/or summer birth deficit, while the similar non-significant tendencies were also observed in the other subgroups.

Until now, Polanczyk et al. [13] is the only study which investigated the season of birth effect on PLEs, to our knowledge. In their study using 2,232 British children of age 12 years, the odds ratio of winter–spring birth for the presence of PLEs was 1.3 (95 % CI = 0.8–1.9), while the statistical level was not significant (adjusted *p* = 0.28, unadjusted *p* by Chi-square test = 0.18).The present study, using larger number of adolescent subjects (*n* = 17,702), clearly showed the significant association between winter birth and PLEs. The definition of summer/winter seasons seems to be reliable because we defined them on the basis of the meteorological data and they were consistent with the conventional definition consequently. Considering share of an extensive range of risk factors between PLEs and schizophrenia [12], our findings may further support construct validity between the clinical and subclinical phenotypes of schizophrenia. The non-significant relationship with winter–spring birth in the previous study [13] may result from the lack of statistical power.

Several limitations may be considered before interpreting the present results. First, PLEs in the present study were assessed by self-rating questionnaires. The validity of self-reported PLEs has not been fully established; therefore, self-reported PLEs might not be equated with that in the original conceptualization [18]. Actually, PLEs were assessed by structured interviews by a child psychiatrist in the longitudinal study, which showed a continuum between PLEs in childhood and schizophrenia spectrum disorder in adulthood [10]. In that study [10], the prevalence of definite PLEs (1.6 %) was significantly lower than that in the present study (14.3 %), while some interview-based studies did not show very low prevalence (for instance, 6.6 % in Kelleher et al. [19]). Second, the clinical relevance of PLEs in childhood and their predictive validity for later development of psychosis and other mental disorders remains to be further studied [11]. While we found PLEs might be associated with suicidal risk and violent behaviors in the same cross-sectional survey data [20, 21], longitudinal studies may be needed to understand the characteristics of the subjects with PLEs. Third, we did not take into consideration the confounding effect of genetic or other environmental factors, including social class, urban birth, and obstetric complications [12]. It may be also interesting to analyze combined effect of the seasonality of birth and these other factors on PLEs because winter birth effect is small. Forth, we could not obtain answers from absent students. Poor mental health status and psychopathology may be more prevalent among frequent or long-term absentees.

In conclusion, PLEs, subclinical correlates of schizophrenia, may also be affected by birth season. Further investigation of this phenomenon may be recommended to elucidate the still unknown etiopathological mechanisms of schizophrenia and establish strategies for its prevention.

## Electronic supplementary material

Below is the link to the electronic supplementary material.

Supplementary material 1 (PDF 156 kb)
