# Supplementary material for: Season of birth effect on psychotic-like experiences in Japanese adolescents
Source: Eur Child Adolesc Psychiatry. 2012 Sep 17;22(2):89–93. doi: 10.1007/s00787-012-0326-1 (PMC3562433; doi:10.1007/s00787-012-0326-1)

Electronic Supplementary Material
European Child & Adolescent Psychiatry

Season of birth effect on psychotic-like experiencesin Japanese adolescents

Mamoru Tochigi[1)], Atsushi Nishida[2)], Shinji Shimodera[3)*], Yuji Okazaki[5)], Tsukasa Sasaki[6)]

1) Department of Neuropsychiatry, Graduate School of Medicine, University of Tokyo, , Tokyo, Japan
2) Department of Psychiatry and Behavioral Sciences, Tokyo Metropolitan Institute of Medical Science, Tokyo, Japan
3) Department of Neuropsychiatry, Kochi Medical School, Kochi, Japan
4) Tokyo Metropolitan Matsuzawa Hospital, Tokyo, Japan
5) Department of Health Education, Graduate School of Education and Office for
Mental Health Support, University of Tokyo, Tokyo, Japan

Address correspondence to; Shinji Shimodera E-mail:shimodes@kochi-u.ac.jp

Supplementary Table 1. The average highest and lowest temperature in the past 20 years (1981-2000) in Kochi and Mie prefectures

	Jan	Feb	Mar	Apr	May	Jun	Jul	Aug	Sep	Oct	Nov	Dec
Kochi												
Highest	12.0	11.1	15.6	20.3	24.1	26.0	30.4	31.4	29.1	24.9	18.8	14.2
Lowest	1.4	1.5	5.8	10.6	15.4	19.4	23.4	24.0	21.5	14.9	9.1	4.4
Mie												
Highest	9.4	9.2	12.5	18.2	22.1	24.6	30.1	30.9	26.9	22.4	17.3	11.8
Lowest	2.5	1.9	5.3	10.3	15.0	19.2	24.0	24.2	20.9	15.0	9.9	4.6

* The temperatures are described in degrees Celsius.

Supplementary Table 2. The odds ratios of winter/summer birth for the prevalence of PLEs by gender, age (junior/senior high school), and survey area

	Season	At least one type of PLEs	"Heard voice"	"Spied upon"
Gender				
Male	Winter	1.08 (0.95-1.24)	1.15 (0.99-1.34)	1.10 (0.90-1.35)
	Summer	1.00 (0.86-1.16)	0.85 (0.71-1.01)	1.07 (0.85-1.35)
Female	Winter	1.13 (1.01-1.27)*	1.09 (0.95-1.24)	1.09 (0.94-1.27)
	Summer	0.88 (0.77-1.00)*	0.92 (0.79-1.07)	0.84 (0.70-1.00)
High school				
Junior	Winter	1.14 (1.02-1.29)*	1.20 (1.04-1.37)**	1.07 (0.90-1.27)
	Summer	0.96 (0.84-1.09)	0.87 (0.74-1.01)	0.97 (0.80-1.17)
Senior	Winter	1.08 (0.96-1.22)	1.03 (0.89-1.20)	1.12 (0.94-1.33)
	Summer	0.89 (0.77-1.03)	0.91 (0.77-1.08)	0.87 (0.71-1.06)
Survey area				
Kochi	Winter	1.03 (0.93-1.15)	1.01 (0.90-1.15)	1.04 (0.90-1.21)
	Summer	0.93 (0.82-1.04)	0.93 (0.81-1.07)	0.90 (0.76-1.07)
Mie	Winter	1.31 (1.13-1.54)***	1.37 (1.14-1.64)***	1.27 (1.02-1.58)*
	Summer	0.93 (0.78-1.11)	0.78 (0.63-0.97)*	0.93 (0.73-1.19)

Odds ratios were calculated by comparing winter vs. summer and other months, or summer vs. winter and other months (described with 95% CI in the brackets).

* $P < 0.05$, ** $P < 0.02$, *** $P < 0.001$

Supplementary Figure 1. The distributions of the prevalence of the four PLEs by birth months

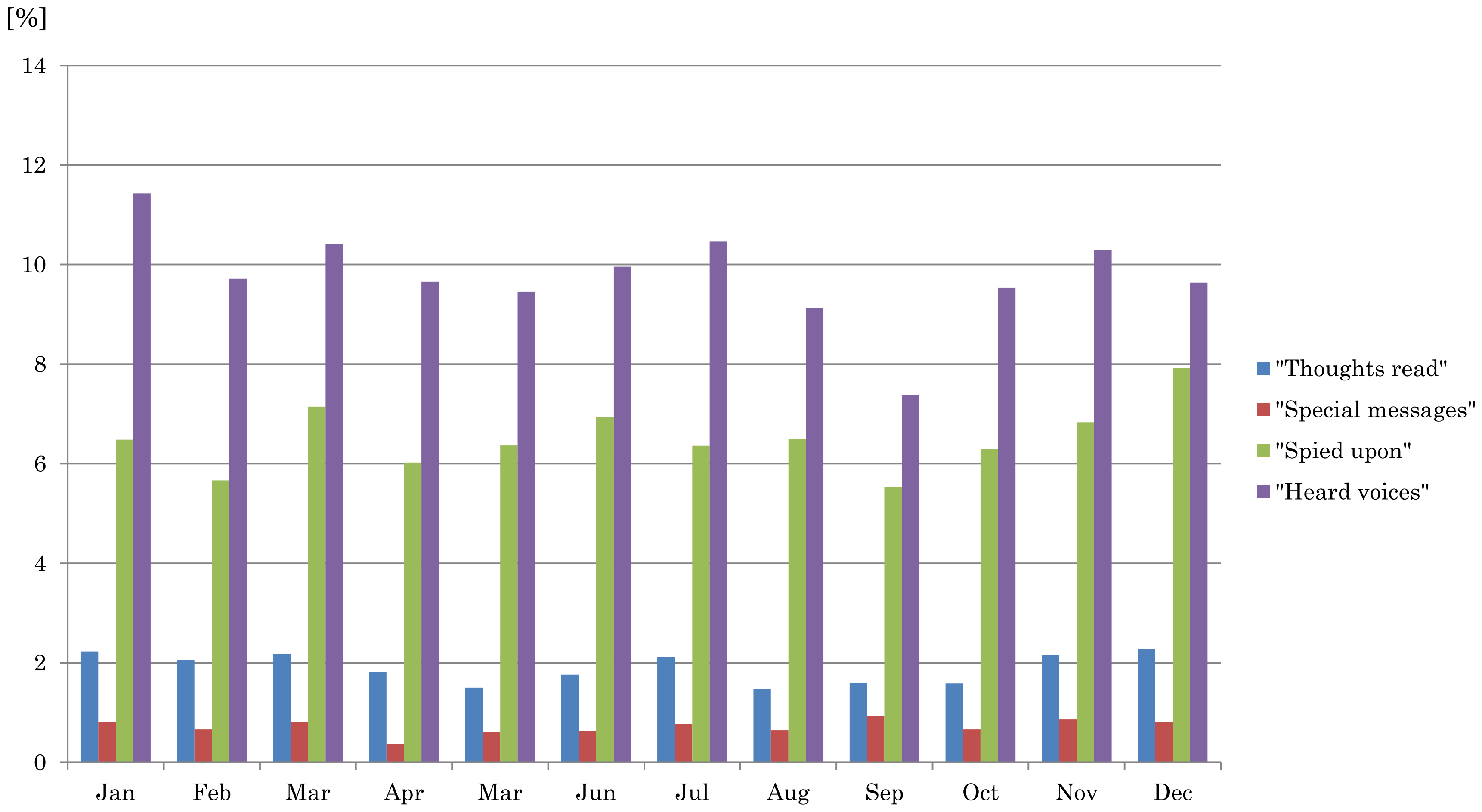

Supplement: Supplementary file 1 — Supplementary material 1 (PDF 156 kb) [file 787_2012_326_MOESM1_ESM.pdf]
